# Psychometric properties of the test of nonverbal intelligence–third edition (TONI-3) in Turkish middle school students

**DOI:** 10.3389/fpsyg.2026.1828497

**Published:** 2026-09-03

**Authors:** Ahmet Bildiren, Sevinç Zeynep Kavruk

**Affiliations:** 1 Department of Special Education, Faculty of Education, Aydın Adnan Menderes University, Aydın, Türkiye; 2 Department of Child Development, Faculty of Health Sciences, Aydın Adnan Menderes University, Aydın, Türkiye

**Keywords:** adolescent, intelligence tests, psychometrics, students, TONI-3

## Abstract

**Background:**

The aim of this study was to examine the psychometric properties of Form A of the Test of Nonverbal Intelligence–Third Edition (TONI-3) in Turkish middle school students aged 10 years 0 months to 14 years 0 months. Although TONI-3 has previously been validated in Turkish children and late adolescents, psychometric evidence for early adolescence remains limited.

**Method:**

The sample consisted of 1,041 middle school students in Aydın province (50.6% female; *M* = 11.74, *SD* = 1.15), selected using stratified cluster sampling based on the Socio-Economic Development Level (SEGE). Internal consistency was assessed using McDonald’s omega total (ωt) and Cronbach’s alpha. Test–retest (*n* = 94), alternate-form reliability (*n* = 114), and criterion-related validity were evaluated using Pearson correlations with the Naglieri Nonverbal Ability Test–I (NNAT-I; *n* = 118) and Raven Standard Progressive Matrices (RSPM; *n* = 105). Group differences were examined using independent-samples *t*-tests and one-way ANOVA.

**Results:**

Internal consistency was high across the examined age, grade, and socioeconomic subgroups (ωt = 0.932; *α* = 0.925). Test–retest reliability (*r* = 0.847) indicated high rank-order stability across administrations despite a small systematic increase in mean scores, and alternate-form reliability was also high (*r* = 0.812). TONI-3 scores showed strong correlations with NNAT-I (*r* = 0.756) and RSPM (*r* = 0.810), supporting criterion-related validity. Confirmatory factor analysis provided partial support for approximate unidimensionality, with favorable CFI (0.983) and RMSEA (0.048), but an elevated SRMR (0.141) and relatively low loadings for introductory basal items. Scores differed significantly across age and grade, whereas the gender difference favored girls with a negligible effect size (*d* = 0.128). No significant differences were observed across socioeconomic development levels.

**Discussion:**

The findings provide complementary evidence supporting the reliability and validity of TONI-3 Form A. Although the structural analyses supported approximate unidimensionality rather than a strictly unidimensional structure, the combined evidence supports the use of TONI-3 Form A for assessing nonverbal intelligence in Turkish middle school students aged 10–14 years, within the scope of the present study. The findings also provide preliminary evidence of comparable item functioning across demographic groups, although formal measurement invariance remains to be established.

## 1 Introduction

Fluid intelligence is a neurologically based cognitive ability that refers to an individual’s capacity to solve novel and unfamiliar problems, discover relationships, and comprehend abstract patterns independently of previously acquired knowledge and experience (Kent, 2017). According to Kaufman and Kaufman (1993), fluid intelligence is defined as the ability to adapt to new problems and generate solutions; within Cattell’s (1971) theoretical framework, it is conceptualized as one of the fundamental cognitive resources supporting the development of crystallized intelligence. However, fluid intelligence is not limited to abstract reasoning alone; rather, it represents a multi-component construct operating through interactions with attention, working memory, and executive functions (Salthouse et al., 2008). This multidimensional nature necessitates the use of assessment tools that minimize reliance on learned knowledge, linguistic proficiency, and cultural familiarity when measuring fluid intelligence. In this context, nonverbal intelligence tests have been developed to obtain more robust indicators of fluid reasoning. Structured around geometric shapes, pattern completion tasks, and visuospatial relationships devoid of verbal content, these instruments aim to reduce biases arising from differences in language proficiency, educational background, and cultural experience (Bracken and McCallum, 2009; Brown, 2003; Moore et al., 2017; McCallum et al., 2001). An integrative review by Orellana covering the years 2013–2023 also demonstrates that intelligence quotient (IQ) alone is insufficient to represent human cognitive capacity, highlighting that nonverbal intelligence in particular reflects a multidimensional and context-sensitive construct (Orellana, 2025). Within this framework, nonverbal intelligence should be considered not merely a subdimension of general intelligence but an essential component of cognitive performance.

It is well established that verbal assessments may inadequately or inaccurately reflect an individual’s cognitive capacity, particularly in contexts where linguistic and cultural differences are pronounced. Consequently, efforts to develop culturally fair assessment tools constitute an important focus of psychometric research (Ping, 2024). By minimizing reliance on linguistic content and academic knowledge, nonverbal tests aim to measure an individual’s real-time reasoning capacity rather than their repertoire of learned knowledge (Johnsen, 2017). In doing so, they provide a more inclusive assessment approach, particularly for multicultural, immigrant, disadvantaged, or linguistically diverse populations.

The distinctiveness of nonverbal intelligence is not limited to its measurement context. Studies examining its relationship with language abilities indicate that nonverbal cognitive processes play a central role in the cognitive foundations of academic performance. Lázaro-Ibarrola (2024) reported that nonverbal intelligence is positively associated with receptive and productive language skills other than speech, while Rosselli et al. (2016) found that individual differences in cognitive performance are primarily determined by nonlinguistic reasoning and visuospatial processing capacity rather than language experience. Research conducted in the context of second language learning further indicates that nonverbal intelligence is a significant predictor in cognitively demanding domains such as reading comprehension and the acquisition of grammatical structures (Kakhramonov, 2020). These findings suggest that nonverbal intelligence is not entirely independent of academic language performance; rather, it constitutes a foundational cognitive capacity underlying academic achievement. The distinction between nonverbal and verbal intelligence is also supported at the neurodevelopmental level. Khundrakpam et al. (2017) reported that these two cognitive domains exhibit different patterns in terms of brain structural covariance networks, suggesting that while verbal intelligence is associated with more stable structural network organizations, nonverbal intelligence may be linked to more dynamic functional processes. This distinction highlights the unique role of nonverbal intelligence, particularly in situations requiring novel problem solving that does not rely on previously acquired knowledge.

Different developmental and clinical samples have also highlighted the distinctive importance of nonverbal intelligence. In individuals with autism, discrepancies between nonverbal intelligence and emotion recognition abilities have been shown to significantly predict difficulties in empathy and socio-emotional functioning (Hadjikhani et al., 2023). In children and adolescents diagnosed with anorexia nervosa, full-scale IQ scores are typically within the normal range, yet specific reductions in nonverbal reasoning components have been observed (Kjaersdam Telléus et al., 2015). Similarly, in children with dyslexia, cognitive difficulties have been linked to impairments in nonverbal serial ordering and temporal sequencing processes (Cowan et al., 2017). In addition, nonverbal intelligence has been shown to demonstrate significant associations with domains such as musical ability (Barrientos-Fernández et al., 2019; Swaminathan et al., 2017), computational thinking (Boom et al., 2018), and early coding skills (Marinus et al., 2018). Taken together, this multidisciplinary body of evidence indicates that nonverbal intelligence plays a central role not only in academic contexts but also across domains that require cognitive flexibility, pattern recognition, and problem solving.

One of the instruments commonly used to assess nonverbal intelligence is the Test of Nonverbal Intelligence–Third Edition (TONI-3). Structured around universal geometric shapes and abstract patterns, TONI-3 is widely used in the assessment of fluid reasoning due to its format that does not require linguistic content (Gallinat and Spaulding, 2014). Studies conducted in Türkiye have demonstrated that TONI-3 is highly valid and reliable for children aged 6–11 years and shows strong correlations with the Raven Progressive Matrices and the Wechsler performance subtests (Korkmaz et al., 2018). Similarly, studies involving gifted children have reported high internal consistency and criterion-related validity coefficients (Bildiren and Korkmaz, 2018). More recent findings indicate that TONI-3 also demonstrates strong psychometric properties during adolescence (14–17 years), showing significant associations with the Raven Standard Progressive Matrices and the Naglieri Nonverbal Ability Test (Bildiren et al., 2024). Taken together, these findings suggest that TONI-3 is a reliable instrument for assessing fluid intelligence during childhood and late adolescence. However, studies conducted in Türkiye have largely focused on children aged 6–11 years and adolescents aged 14–17 years, and no study has systematically examined the psychometric properties of TONI-3 specifically in children aged 10 years 0 months to 14 years 0 months, an important developmental period bridging late childhood and early adolescence. The middle school period is characterized by substantial cognitive development, during which improvements in cognitive flexibility, fluid reasoning, and executive functions have been reported in previous developmental research (Blake and Pope, 2008; Buttelmann and Karbach, 2017; Ferguson et al., 2021; Madanagopal, 2020). This period has also been associated with important changes in the organization of cognitive processes (Cherukunnath and Singh, 2022; Demetriou et al., 2023). During such developmental transition periods in which cognitive processes are being reorganized, it cannot be assumed that the psychometric properties of measurement instruments remain stable. Given previous evidence that fluid intelligence is associated with developmental changes in working memory capacity, processing speed, and executive functioning (Schulz-Zhecheva et al., 2024; Swanson, 2008; Tourva et al., 2016; Tourva and Spanoudis, 2020), it is important to evaluate whether the psychometric properties of the instrument remain stable during this developmental period. Accordingly, the present study aims not only to address the lack of empirical data for this age range but also to systematically evaluate the reliability, validity, and internal structure of TONI-3 Form A during early adolescence.

Although adjacent investigations in Türkiye indicate that the TONI-3 is a valid and reliable instrument for children aged 6–11 years (Korkmaz et al., 2018) and late adolescents aged 14–17 years (Bildiren et al., 2024), the middle school period (10 years 0 months to 14 years 0 months) has not yet been examined as a distinct developmental group, leaving an important gap in the available psychometric evidence. Reliable, low-language-load measures of nonverbal intelligence are particularly important during the cognitive and educational transitions of early adolescence. Accordingly, the present study aims to systematically evaluate the reliability, structural validity, and criterion-related validity of TONI-3 Form A among Turkish middle school students. Beyond extending the available evidence to this age group, the present study provides a more comprehensive psychometric evaluation than previous Turkish validation studies by integrating classical test theory indices with confirmatory factor analysis, Rasch model estimation, differential item functioning (DIF) analyses, and standard error of measurement (SEM) estimates. These analyses provide complementary evidence regarding the instrument’s internal structure, measurement precision, and item functioning during early adolescence.

## 2 Materials and methods

### 2.1 Participants

The population of the study consisted of 57,213 middle school students enrolled in Aydın province (Ministry of National Education, 2025). The sample was determined based on the Socio-Economic Development Ranking Research (SEGE) index published by the Ministry of Industry and Technology of the Republic of Türkiye. SEGE classifies provinces and districts according to their levels of socioeconomic development using various indicators, including education, health, finance, and infrastructure. Based on SEGE index scores and the Jenks Natural Breaks classification method, the districts of Aydın province were categorized into four different levels of socioeconomic development (Ministry of Industry and Technology, 2022).

To obtain a sample that represents the province and reflects different socioeconomic structures, a stratified cluster sampling method was employed. Within this framework, each socioeconomic development level was treated as a separate stratum, and at least one school from each stratum was included in the sample. Three schools were selected from the first level, where population density was higher, while one school was selected from each of the second, third, and fourth levels. Schools were treated as cluster units, and eligible participants were students ranging in age from 10 years 0 months to 14 years 0 months who were enrolled in the relevant grade levels. During the sampling process, care was taken to ensure proportional representation of gender and grade levels within each SEGE category. Consequently, the final sample represented the different socioeconomic levels of the province.

A total of 1,041 children participated in the study. The demographic characteristics of the participants are presented in Table 1.

**Table 1:** Demographic characteristics of the participants.

Variables	*n* (%)
Gender	
Female	527 (50.6%)
Male	514 (49.4%)
Children’s age, *M* ± SD	11.74 ± 1.15
Age (years, months)	
10y 0 m – 10y 11 m	203 (19.5%)
11y 0 m – 11y 11 m	255 (24.5%)
12y 0 m – 12y 11 m	282 (27.1%)
13y 0 m – 14y 0 m	301 (28.9%)
Grade	
5th grade	285 (27.4%)
6th grade	227 (21.8%)
7th grade	271 (26.0%)
8th grade	258 (24.8%)
Socioeconomic Development Level (SEGE)	
1st Level	504 (48.4%)
2nd Level	230 (22.1%)
3rd Level	162 (15.6%)
4th Level	145 (13.9%)

Table 1 presents the demographic characteristics of the study sample. Of the participants, 50.6% were female (*n* = 527) and 49.4% were male (*n* = 514). The mean age was 11.74 years (*SD* = 1.15), with participants ranging in age from 10 years 0 months to 14 years 0 months. The largest proportion of participants belonged to the 13-year-old age group (28.9%). The distribution across grade levels was relatively balanced, with the highest proportion observed among 5th-grade students (27.4%). In terms of socioeconomic development levels (SEGE), the largest proportion of participants was classified as Level 1 (48.4%).

### 2.2 Measurements

*The Test of Nonverbal Intelligence–Third Edition (TONI-3)*, developed by Brown et al. (1997), is a nonverbal intelligence test designed to assess general cognitive ability in individuals aged 6–89 years while minimizing the influence of language. TONI-3 Form A consists of 45 dichotomously scored items arranged in increasing order of difficulty. Each correct response receives one point and each incorrect response receives zero points, yielding a total raw score ranging from 0 to 45. The Turkish validation and standardization study of TONI-3 was conducted by Korkmaz et al. (2018) with a sample of 631 children aged 6–11 years. The study reported KR-20 internal consistency coefficients ranging from 0.86 to 0.95 for Form A and from 0.90 to 0.93 for Form B. In addition, strong positive correlations were found between TONI-3 and the Raven Standard Progressive Matrices (RSPM) (*r* = 0.79 for Form A and r = 0.82 for Form B), providing evidence for the criterion-related validity of the Turkish version. In another study conducted with 566 adolescents aged 14–17 years, the average reliability coefficient of the test was reported as 0.92, the correlation between Form A and Form B was high (*r* = 0.76), and the test–retest reliability calculated over a four-week interval was 0.83. In the same study, the strong and significant correlations between TONI-3 scores and both the RSPM and the Naglieri Nonverbal Ability Test–I (NNAT-I) supported the validity and reliability of the test for the adolescent age group as well (Bildiren et al., 2024). In the present study, Form A of TONI-3 was used. Reliability (internal consistency, test–retest reliability, and alternate-form reliability) and criterion-related validity analyses for Form A were conducted using a sample of participants ranging in age from 10 years 0 months to 14 years 0 months.

*The Naglieri Nonverbal Ability Test–I (NNAT-I)*, developed by Naglieri, is a nonverbal assessment instrument designed to measure general mental ability, which is considered a precursor to academic achievement (Naglieri, 2003). Similar to other general ability tests, NNAT-I aims to evaluate an individual’s overall cognitive capacity and consists of 72 matrix items. Items are scored using a binary scoring system (1 = correct, 0 = incorrect).

The original norm study of the test was conducted in the United States with 1,585 individuals aged 5–17 years. The Cronbach’s alpha coefficient of the test was reported as 0.91, and the test–retest reliability coefficient was 0.71. As part of the construct validity analyses, the correlation with the Matrix Analogy Test was reported to be approximately 0.74. In addition, correlations of 0.78 between NNAT and the Standard Progressive Matrices Test, 0.63 between NNAT-I and the Test of Nonverbal Intelligence–Third Edition (TONI-3), and correlations ranging from 0.31 to 0.65 between NNAT-I and the WISC-IV (Wechsler Intelligence Scale for Children–Fourth Edition) were reported (Naglieri, 2003). In the present study, only raw scores from NNAT-I were used, and these raw scores were directly used in the correlation analyses conducted for validity purposes.

*The Raven Standard Progressive Matrices (RSPM)*, developed by Raven (1983), is a nonverbal instrument designed to assess intelligence. The test consists of 60 items organized into five sets (A–E), each containing 12 items. The items consist of geometric patterns with a missing component, and participants are required to select the correct piece from six or eight alternatives to complete the pattern. The difficulty level of the items increases progressively both within and across sets, primarily through an increase in the number of response options and the complexity of the patterns. Each correct response is scored as one point. The test can be administered across a wide age range, from 6 years of age to adulthood. The adaptation and standardization study of the RSPM for Turkish culture was conducted by Şahin and Düzen with 2,277 children aged 6–15 years. In that study, the split-half reliability coefficient of the test was reported as 0.91 for the overall sample. Correlations among the subtests ranged between 0.58 and 0.95, supporting both the construct validity and internal consistency of the scale (Şahin and Düzen, 1994). In the present study, raw RSPM scores were used and these scores were included in the criterion-related validity analyses conducted with TONI-3.

### 2.3 Procedure

Prior to the implementation of the study, ethical approval was obtained from the Education Research Ethics Committee of Aydın Adnan Menderes University (Approval No. 2025/10, dated 30 October 2025). Following the ethics approval, official permission to conduct data collection in schools was obtained from the Ministry of National Education. After the completion of the permission procedures, school administrations were contacted and the application schedule was arranged so as not to disrupt regular school activities in the randomly selected schools.

Before the data collection process, written informed consent was obtained from the parents of the participating students. Test administrations were conducted in a group format by the second author, a specialist in child development, following the standardized administration procedures of the TONI-3. A quiet testing environment was ensured, and the first five sample items were completed collectively with the class to ensure that the instructions were fully understood. After the sample items, students responded to the remaining items individually. The administration lasted approximately 30 min. Because the test was administered in a group format, all participants were instructed to respond to all 45 items to ensure a standardized administration procedure. However, final test scores were calculated individually in accordance with the standardized TONI-3 discontinue rule; therefore, responses beyond each participant’s discontinue point were excluded from the calculation of the total score. In contrast, all observed item responses were retained for item-level psychometric analyses, including reliability analyses, item difficulty, corrected item–total correlations, CFA, Rasch calibration, EAP person reliability, and DIF analyses.

Additional applications were conducted to examine the validity and reliability of the test. In this context, a test–retest administration was conducted with 94 children to determine score stability, and an alternate-form administration was conducted with 114 children to evaluate form equivalence. For the criterion-related validity analysis, the Raven Standard Progressive Matrices Test (RSPM) was administered to 105 children and the Naglieri Nonverbal Ability Test–I (NNAT-I) was administered to 118 children.

### 2.4 Statistical analysis

Statistical analyses were conducted using IBM SPSS Statistics version 25, JASP version 0.9.5.4.0, and R version 4.5.2 (R Core Team, 2025). Frequencies and percentages were calculated to describe the demographic characteristics of the participants. The assumption of normality was evaluated using skewness and kurtosis coefficients. For the total TONI-3 scores, the skewness coefficient was −0.107 (SE = 0.076) and the kurtosis coefficient was −0.472 (SE = 0.151). These values fell within the ±1 range. In addition, the closeness of the mean (*M* = 27.31), median (27.00), and 5% trimmed mean (27.39) supported the symmetric distribution of the data. Based on these findings, the use of parametric tests was considered appropriate.

The reliability of the measurement instrument was examined using the Pearson product–moment correlation coefficient (r) for test–retest and alternate-form reliability. Internal consistency was evaluated using Cronbach’s alpha (*α*) and McDonald’s omega total (*ω*t). Because TONI-3 Form A consists of dichotomously scored items, Cronbach’s alpha is mathematically equivalent to the Kuder–Richardson Formula 20 (KR-20) reported in the original standardization study. Cronbach’s alpha was retained to facilitate comparison with McDonald’s omega total (ωt), which was estimated under the unidimensional factor model examined in the present study. For item-level psychometric analyses, including internal consistency, item difficulty, corrected item–total correlations, CFA, Rasch calibration, EAP person reliability, and DIF analyses, all observed item responses were retained, regardless of each participant’s discontinue point. In contrast, the standardized TONI-3 discontinue rule was applied only when calculating participants’ final total scores. Accordingly, item-level psychometric analyses and total-score analyses were based on different scoring conventions, consistent with their distinct analytical purposes. Retaining all observed responses provided complete item-level information and avoided the ability-dependent missingness that would otherwise have resulted from applying the discontinue rule to item-level analyses. To provide an index of score precision for individual assessment, the standard error of measurement (SEM) was calculated from McDonald’s omega using the formula SEM = SD × √(1 − ω), where SD represents the standard deviation of the final TONI-3 total scores obtained according to the standardized discontinue rule.

To evaluate the internal structure of the instrument, a confirmatory factor analysis (CFA) was conducted in R (version 4.5.2) using the cfa() function in the *lavaan* package (version 0.6–21). The dichotomously scored TONI-3 items were treated as ordered categorical variables and estimated using the diagonally weighted least squares (DWLS) estimator with robust standard errors and a mean- and variance-adjusted (scaled-shifted) chi-square test statistic. The CFA was specified as a one-factor model to evaluate the hypothesized unidimensional structure of TONI-3 Form A. Because Item 1 was answered correctly by all participants (100% correct response rate), resulting in zero variance, it was excluded from the CFA, and the analysis was conducted on Items 2–45. Model fit was evaluated using the chi-square statistic (*χ*^2^), the Comparative Fit Index (CFI), the Tucker–Lewis Index (TLI), the Root Mean Square Error of Approximation (RMSEA) with its 90% confidence interval, and the Standardized Root Mean Square Residual (SRMR). Following the recommendations of Hu and Bentler (1999), CFI and TLI values of 0.90 or greater and RMSEA and SRMR values of 0.08 or lower were considered indicative of acceptable model fit. For the ordinal CFA, threshold parameters were also estimated, and the threshold estimates for all analyzed items are presented in Supplementary Table S1. In addition, local dependence was evaluated by examining standardized residual correlations between item pairs. Standardized residual correlations with absolute values ≥ 0.30 were considered potential indicators of local dependence and are reported in Supplementary Table S2. The scaled chi-square statistic and degrees of freedom are reported as returned by the lavaan package. Because a corresponding chi-square *p*-value was not available, interpretation of model fit focused primarily on the approximate fit indices (CFI, TLI, RMSEA, and SRMR).

To complement the CFA findings, item-level psychometric analyses were conducted to evaluate item functioning and measurement precision. Item difficulty indices (proportion correct) and corrected item–total correlation coefficients were calculated to assess item characteristics and discrimination. Subsequently, Rasch analysis based on the one-parameter logistic (1PL) model was performed to estimate item difficulty, evaluate item fit using infit and outfit mean square (MNSQ) statistics, and estimate person reliability using Expected A Posteriori (EAP) reliability. Because Item 1 exhibited no response variability, Rasch calibration yielded an extreme difficulty estimate for this item. Therefore, its difficulty estimate is reported in Supplementary Table S1 for completeness but was not considered substantively interpretable. Consistent with recommendations in the Rasch measurement literature, greater emphasis was placed on infit statistics because they are less sensitive than outfit statistics to unexpected responses on items located far from an individual’s estimated ability level (Bond and Fox, 2015).

To examine whether items functioned similarly across demographic groups, differential item functioning (DIF) analyses were conducted using a multiple-group Rasch item response theory model. Because Item 1 exhibited no response variability, it was excluded from the DIF analyses. Consequently, DIF analyses were performed on the remaining 44 items (Items 2–45) and examined separately across gender and age (10 years 0 months to 14 years 0 months) groups. For the binary gender comparison, Rasch item difficulty contrasts between groups and the direction of DIF (i.e., the group for which an item was relatively easier or more difficult) were additionally examined to evaluate the practical magnitude of potential differential item functioning. Because age was evaluated using a four-group omnibus Rasch model, only likelihood-ratio chi-square statistics were reported for the age analyses. To control for multiple comparisons, *p*-values were adjusted using the Benjamini–Hochberg false discovery rate procedure.

For validity analyses, the relationships between TONI-3, Raven Standard Progressive Matrices Test (RSPM), and Naglieri Nonverbal Ability Test–I (NNAT-I) scores were evaluated using Pearson correlation analyses. An independent samples *t*-test was conducted to determine whether TONI-3 scores differed by gender. Group differences according to age, grade level, and socioeconomic development level were examined using one-way analyses of variance (ANOVA). Prior to ANOVA, homogeneity of variances was evaluated using Levene’s test. When the assumption was satisfied, Tukey’s honestly significant difference (HSD) test was used for *post hoc* comparisons. For each ANOVA, eta-squared (*η*^2^) effect sizes were calculated and interpreted according to conventional benchmarks (0.01 = small, 0.06 = medium, 0.14 = large).

## 3 Results

Table 2 shows the descriptive statistics of the study variables according to gender, age group, grade level, and socioeconomic development level (SEGE).

**Table 2:** Descriptive statistics of the study variables.

Variables	*n*	M ± SD	Min	Max
Gender				
Female	527	27.79 ± 7.45	6.00	45.00
Male	514	26.81 ± 7.83	7.00	44.00
Age (years, months)				
10y 0 m – 10y 11 m	203	24.54 ± 6.90	7.00	42.00
11y 0 m – 11y 11 m	255	26.02 ± 7.09	7.00	43.00
12y 0 m – 12y 11 m	282	28.58 ± 7.71	6.00	43.00
13y 0 m – 14y 0 m	301	29.07 ± 7.84	9.00	45.00
Grade				
5th grade	285	24.30 ± 7.10	7.00	43.00
6th grade	227	26.61 ± 6.94	7.00	43.00
7th grade	271	29.20 ± 7.68	6.00	44.00
8th grade	258	29.26 ± 7.68	9.00	45.00
Socioeconomic Development Level (SEGE)				
1st Level	504	27.18 ± 7.74	7.00	45.00
2nd Level	230	28.23 ± 7.71	9.00	44.00
3rd Level	162	27.49 ± 7.41	6.00	43.00
4th Level	145	26.08 ± 7.43	9.00	43.00

Table 2 shows that mean TONI-3 scores were higher for girls (*M* = 27.79, *SD* = 7.45) than for boys (*M* = 26.81, *SD* = 7.83). Across age groups, mean scores increased with age, with the lowest mean observed in the 10-year-old group (*M* = 24.54, *SD* = 6.90) and the highest mean in the 13-year-old group (*M* = 29.07, *SD* = 7.84). A similar pattern was observed across grade levels, with mean scores increasing from Grade 5 to Grade 8. The lowest mean was observed in Grade 5 (*M* = 24.30, *SD* = 7.10), whereas the highest mean was observed in Grade 8 (*M* = 29.26, *SD* = 7.68). Mean scores across SEGE levels were relatively similar, with the highest mean in Level 2 (*M* = 28.23, *SD* = 7.71) and the lowest mean in Level 4 (*M* = 26.08, *SD* = 7.43). The minimum and maximum test scores were similar across all groups.

Table 3 presents the reliability coefficients of the test, including McDonald’s omega (*ω*) and Cronbach’s alpha (*α*), according to age, grade level, and socioeconomic development level.

**Table 3:** McDonald’s omega, Cronbach’s alpha, 95% confidence intervals, and standard error of measurement according to age, grade, and socioeconomic development level.

Variables	McDonald’s *ω* (95% CI)	SEM	Cronbach’s α (95% CI)
Age (years, months)			
10y 0 m – 10y 11 m	0.925 (0.91–0.94)	1.89	0.914 (0.899–0.928)
11y 0 m – 11y 11 m	0.921 (0.907–0.935)	1.99	0.913 (0.898–0.925)
12y 0 m – 12y 11 m	0.934 (0.922–0.945)	1.98	0.925 (0.914–0.936)
13y 0 m – 14y 0 m	0.936 (0.925–0.946)	1.98	0.929 (0.918–0.938)
Total	0.932 (0.926–0.938)	1.99	0.925 (0.919–0.931)
Grade			
5th grade	0.927 (0.915–0.939)	1.92	0.917 (0.904–0.928)
6th grade	0.915 (0.899–0.931)	2.02	0.910 (0.894–0.924)
7th grade	0.933 (0.922–0.945)	1.99	0.924 (0.912–0.935)
8th grade	0.932 (0.920–0.944)	2.00	0.926 (0.914–0.937)
Total	0.932 (0.926–0.938)	1.99	0.925 (0.919–0.931)
Socioeconomic Development Level (SEGE)			
1st Level	0.933 (0.925–0.941)	2.00	0.926 (0.918–0.934)
2nd Level	0.931 (0.918–0.944)	2.03	0.925 (0.912–0.937)
3rd Level	0.930 (0.914–0.945)	1.96	0.921 (0.904–0.936)
4th Level	0.931 (0.915–0.947)	1.95	0.927 (0.910–0.941)
Total	0.932 (0.926–0.938)	1.99	0.925 (0.919–0.931)

CI = confidence interval; SEM = standard error of measurement. SEM values were calculated from McDonald’s omega using the formula SEM = SD × √(1 − ω).

Table 3 presents McDonald’s omega and Cronbach’s alpha coefficients together with their 95% confidence intervals and standard error of measurement (SEM) values across age, grade, and socioeconomic development level (SEGE) groups. Internal consistency was consistently high across all subgroups. Across age groups, McDonald’s omega coefficients ranged from 0.921 to 0.936, whereas Cronbach’s alpha coefficients ranged from 0.913 to 0.929. Similar reliability estimates were observed across grade levels (ω = 0.915–0.933; α = 0.910–0.926) and SEGE groups (ω = 0.930–0.933; α = 0.921–0.927). The corresponding 95% confidence intervals were narrow and largely overlapped across subgroups, suggesting no meaningful differences in internal consistency. SEM values ranged from 1.89 to 2.03 across the examined subgroups, indicating a consistently low level of measurement error. For the total sample, the overall reliability coefficients were McDonald’s ωt = 0.932 (95% CI [0.926, 0.938]) and Cronbach’s *α* = 0.925 (95% CI [0.919, 0.931]), with an SEM of 1.99.

Table 4 presents the results of the confirmatory factor analysis (CFA) conducted to evaluate the unidimensional structure of the TONI-3 Form A.

**Table 4:** Confirmatory factor analysis fit indices for the unidimensional model of TONI-3 Form A.

Model	*χ* ^2^	df	CFI	TLI	RMSEA	90% CI for RMSEA	SRMR
One-factor model	3053.41	902	0.983	0.982	0.048	0.046–0.050	0.141

CFA was conducted in R (version 4.5.2) using the cfa() function in the lavaan package (version 0.6–21). Because the TONI-3 items were dichotomously scored, they were treated as ordered categorical variables and estimated using the DWLS (WLSMV) estimator. Item 1 was answered correctly by all participants and therefore showed zero variance; consequently, it was excluded from the CFA, and the analysis was conducted on Items 2–45. The scaled chi-square statistic and degrees of freedom are reported as returned by the lavaan package. Because a corresponding chi-square *p*-value was not available, interpretation of model fit focused primarily on the approximate fit indices (CFI, TLI, RMSEA, and SRMR).

As shown in Table 4, the overall pattern of model fit indices provided partial support for the hypothesized one-factor structure of TONI-3 Form A (CFI = 0.983, TLI = 0.982, RMSEA = 0.048, 90% CI [0.046, 0.050]). Although the SRMR value (0.141) exceeded the conventional cutoff of 0.08 (Hu and Bentler, 1999), the remaining fit indices were consistent with the hypothesized unidimensional model. Accordingly, the CFA findings were interpreted as providing mixed evidence for the approximate unidimensionality of TONI-3 Form A rather than definitive confirmation of a strictly unidimensional structure. Item 1 was answered correctly by all participants, resulting in zero variance (100% correct response rate). Consequently, Item 1 was excluded from the CFA and DIF analyses because its parameters could not be estimated reliably. Its extreme Rasch difficulty estimate is reported in Supplementary Table S1 for completeness. Standardized factor loadings and threshold values for all analyzed items are presented in Supplementary Table S1. As shown in Supplementary Table S1, the introductory basal items (Items 2–11) generally exhibited relatively low standardized factor loadings. Although this pattern is consistent with the extremely high correct response rates and limited response variability expected for very easy basal items, it also indicates that these items contributed relatively little common-factor information in the present middle-school sample. This finding should therefore be considered when interpreting the overall CFA results. Local dependence diagnostics based on standardized residual correlations are presented in Supplementary Table S2.

Table 5 presents the results of the classical item analyses and Rasch analyses for TONI-3 Form A.

**Table 5:** Classical and Rasch-based item-level psychometric evidence for TONI-3 Form A.

Analysis	Statistic	Interpretation
Item Difficulty (p)	0.02–1.00	Items ranged from very easy to very difficult.
Corrected Item–Total Correlation	*M* = 0.42 (Range = 0.02–0.69)	Corrected item–total correlations indicated acceptable discrimination for most items, although several introductory items showed lower values.
Rasch Person Reliability (EAP)	0.937	Excellent measurement precision.
Rasch Item Difficulty (logit)	−6.98 to 5.42 logits (Items 2–45)	Items demonstrated a broad hierarchical difficulty continuum.
Infit Mean Square (MNSQ)	Median = 0.95 (IQR = 0.83–1.14); 38 of 44 estimable items (86.4%) fell within the recommended range (0.70–1.30).	Overall, the majority of estimable items demonstrated acceptable information-weighted fit to the Rasch model.
Outfit Mean Square (MNSQ)	Median = 1.58 (IQR = 0.61–4.24)	Outfit values showed greater variability across introductory and later items. Because outfit statistics are sensitive to unexpected responses far from a respondent’s estimated ability level, they were interpreted cautiously and considered supplementary to infit statistics.

p represents the proportion of correct responses for each dichotomously scored item (0 = incorrect, 1 = correct). Corrected item–total correlations were used as indicators of item discrimination. Rasch item difficulty estimates are reported in logits, and person reliability is reported as Expected A Posteriori (EAP) reliability. Infit and outfit values represent Rasch mean square (MNSQ) fit statistics. Because outfit statistics are particularly sensitive to unexpected responses on items located far from a respondent’s estimated ability level, greater emphasis was placed on infit statistics, which are generally considered more informative for evaluating item fit in Rasch models. Elevated outfit values observed across both introductory and later items were therefore interpreted cautiously rather than as evidence of systematic item misfit.

Table 5 shows that the test included items spanning a broad range of difficulty levels (*p* = 0.02–1.00). Rasch analysis indicated excellent person reliability (EAP = 0.937) and a broad hierarchy of interpretable item difficulty estimates for Items 2–45, ranging from −6.98 to 5.42 logits. Item 1 was retained in the Rasch calibration and in the estimation of EAP person reliability and yielded an extreme difficulty estimate (−40.50 logits) because all participants answered the item correctly; therefore, this estimate was not considered part of the reported meaningful item difficulty range. Overall, the Rasch analysis demonstrated generally acceptable item fit. Based on infit statistics, 38 of the 44 estimable items fell within the commonly recommended interval of 0.70–1.30. Outfit statistics showed greater variability, with elevated values observed across both introductory and later items rather than being confined to the easiest introductory items. Because outfit statistics are particularly sensitive to unexpected responses on items located far from a respondent’s estimated ability level, they were interpreted cautiously and considered supplementary to the infit statistics, which provided the primary basis for evaluating item fit. Detailed item-level statistics, including standardized factor loadings, threshold values, item difficulty estimates, and item fit statistics, are presented in Supplementary Table S1. Item 1 was excluded from the CFA and DIF analyses because all participants answered it correctly (100% correct response rate), resulting in zero variance.

Table 6 presents the results of the differential item functioning (DIF) analyses across gender and age groups.

**Table 6:** Differential item functioning (DIF) analyses across gender and age groups.

Comparison	Item	*χ* ^2^	df	*p*	BH-adjusted *p*	Difficulty contrast (logits)
Gender	Item 6	9.54	1	0.002	0.088	0.59 (M)
	Item 22	5.61	1	0.018	0.214	0.10 (M)
	Item 26	5.07	1	0.024	0.214	0.63 (M)
	Item 27	5.38	1	0.020	0.214	0.64 (M)
	Item 28	5.91	1	0.015	0.214	0.65 (F)
	Item 40	4.09	1	0.043	0.316	0.65 (F)
Age (10 years 0 months to 14 years 0 months)	Item 2	10.53	3	0.015	0.207	
	Item 3	8.16	3	0.043	0.269	
	Item 17	11.12	3	0.011	0.207	
	Item 31	14.77	3	0.002	0.089	
	Item 35	8.64	3	0.035	0.253	
	Item 39	9.79	3	0.020	0.207	
	Item 41	9.49	3	0.023	0.207	

M = relatively easier for males; F = relatively easier for females. DIF contrast represents the absolute difference in Rasch item difficulty estimates between the two gender groups.

*χ*^2^ represents the likelihood-ratio chi-square statistic obtained from the Rasch differential item functioning (DIF) analyses. BH = Benjamini–Hochberg false discovery rate adjusted *p*-value. Item 1 was excluded from the DIF analyses because all participants answered the item correctly, preventing parameter estimation. Although several items demonstrated nominal statistical significance before correction, no items remained statistically significant after Benjamini–Hochberg adjustment. Accordingly, the observed DIF findings should be interpreted with caution and do not provide strong evidence of differential item functioning across the examined groups. For the gender comparison, the difficulty contrast represents the absolute difference in Rasch item difficulty estimates between females and males, and the direction of DIF indicates the group for which the item was relatively easier. Because the age analysis involved four comparison groups, omnibus likelihood-ratio DIF statistics are reported, whereas item-specific difficulty contrasts and DIF direction are presented only for the binary gender comparison.

Table 6 shows that six items demonstrated nominally significant DIF across gender and seven items demonstrated nominally significant DIF across age groups prior to adjustment for multiple comparisons. For the gender comparison, item difficulty contrasts and the direction of DIF were additionally examined to evaluate the practical magnitude of the observed differences. Although several items exhibited small-to-moderate between-group differences in item difficulty, no items remained statistically significant after applying the Benjamini–Hochberg false discovery rate correction. Because the age analysis involved four comparison groups, omnibus likelihood-ratio DIF statistics are reported for age. Overall, these findings provide little evidence of substantial differential item functioning across the examined gender and age groups and should be interpreted with caution.

Table 7 presents the results of the test–retest reliability, alternate-form reliability, and criterion-related validity analyses for TONI-3.

**Table 7:** Test–retest, alternate-form, and criterion-related validity coefficients for TONI-3 Form A.

Analysis type	Measures compared	*n*	*M*	*SD*	*r*	95% CI	ICC	Age-adjusted partial correlation (*r*)	SEM
Test–Retest	Initial Application	94	24.23	5.72	0.847*	[0.778, 0.896]	0.90	–	2.24
	After 4 Weeks		26.07	6.19					
Alternate-form	Form A	114	27.36	7.19	0.812*	[0.738, 0.866]	0.84	–	3.12
	Form B		27.25	6.47					
Criterion Validity	TONI-3	118	29.23	7.85	0.756*	[0.666, 0.824]		0.745	–
	NNAT-I		43.10	10.03					
	TONI-3	105	28.09	7.28	0.810*	[0.732, 0.867]		0.802	–
	RSPM		41.58	9.10					

*All correlation coefficients were statistically significant (*p* < 0.001).

SEM = standard error of measurement. SEM values are reported only for the reliability analyses because SEM is derived from reliability coefficients and is not applicable to criterion-related validity correlations. For the reliability analyses, SEM was calculated as SEM = SD × √(1 − r), where r represents the corresponding reliability coefficient (test–retest or alternate-form reliability).

Table 7 presents the reliability and criterion-related validity results for TONI-3 Form A. Test–retest reliability was high, with a strong correlation between the initial and four-week follow-up administrations (*r* = 0.847, 95% CI [0.778, 0.896], *p* < 0.001) and excellent agreement (ICC = 0.90). Although a small but statistically significant mean difference was observed between administrations (mean difference = −1.84, *t*(93) = −5.37, *p* < 0.001), the corresponding SEM was 2.24, indicating a relatively low level of measurement error. Similarly, alternate-form reliability demonstrated a strong association between Form A and Form B scores (*r* = 0.812, 95% CI [0.738, 0.866], *p* < 0.001) with excellent agreement (ICC = 0.84). No statistically significant mean difference was observed between the two forms (mean difference = 0.12, *t*(113) = 0.31, *p* = 0.758), and the corresponding SEM was 3.12. Criterion-related validity was supported by strong positive correlations between TONI-3 and both the NNAT-I (*r* = 0.756, 95% CI [0.666, 0.824], *p* < 0.001) and the RSPM (*r* = 0.810, 95% CI [0.732, 0.867], *p* < 0.001). After controlling for age, the criterion validity coefficients remained virtually unchanged (partial *r* = 0.745 for NNAT-I and partial *r* = 0.802 for RSPM), indicating that the observed criterion-related validity was not substantially explained by age-related variance.

Table 8 presents the results of the independent samples *t*-test examining gender differences in TONI-3 scores.

**Table 8:** Independent samples *t*-test results for TONI-3 scores by gender.

Group	*n*	*M*	*SD*	*t*	df	*p*	Cohen’s *d*	95% CI
Female	527	27.79	7.45	2.069	1,039	0.039	0.128	[0.007, 0.250]
Male	514	26.81	7.83					

Table 8 shows that female students (*M* = 27.79, *SD* = 7.45) scored significantly higher than male students (*M* = 26.81, SD = 7.83), *t*(1039) = 2.069, *p* = 0.039. The effect size was small (Cohen’s *d* = 0.128, 95% CI [0.007, 0.250]).

Table 9 presents the ANOVA results for TONI-3 total scores according to age, grade level, and socioeconomic development level.

**Table 9:** ANOVA results for TONI-3 total scores by age, grade, and socioeconomic development level.

Variables	Source of the variance	Sum of Squares	df	Mean square	*F*	*p*	*Post hoc* comparisons (Tukey HSD)
Age (years, months)	Between Groups	3376.233	3	1125.411	20.287	*p* < 0.001	10 years < 12 years 10 years < 13 years 11 years < 12 years 11 years < 13 years
	Within Groups	57528.166	1,037	55.476			
	Total	60904.400	1,040				
Grade	Between Groups	4647.548	3	1549.183	28.557	*p* < 0.001	5th grade < 7th grade 5th grade < 8th grade 6th grade < 7th grade 6th grade < 8th grade
	Within Groups	56256.852	1,037	54.250			
	Total	60904.400	1,040				
SEGE Level	Between Groups	429.008	3	143.003	2.452	0.062	None
	Within Groups	60475.392	1,037	58.318			
	Total	60904.400	1,040				

Table 9 shows a significant difference among age groups, *F*(3, 1,037) = 20.287, *p* < 0.001, *η^2^* = 0.055. Post-hoc Tukey HSD tests indicated that the 10- and 11-year-old groups scored significantly lower than the 12- and 13-year-old groups (*p* < 0.05). A significant difference was also observed across grade levels, *F*(3, 1,037) = 28.557, *p* < 0.001, *η^2^* = 0.076. *Post hoc* comparisons showed that Grade 5 and Grade 6 students scored significantly lower than Grade 7 and Grade 8 students (*p* < 0.05). In contrast, no statistically significant differences in TONI-3 scores were observed across socioeconomic development levels (SEGE), *F*(3, 1,037) = 2.452, *p* = 0.062, *η^2^* = 0.007.

## 4 Discussion

The present study examined the psychometric properties of TONI-3 Form A among middle school students ranging in age from 10 years 0 months to 14 years 0 months, thereby providing psychometric evidence for a developmental period that had not previously been examined in the Turkish validation literature. Internal consistency was high across all demographic and socioeconomic subgroups (McDonald’s ωt = 0.932; Cronbach’s *α* = 0.925). Test–retest (*r* = 0.847) and alternate-form (*r* = 0.812) correlations further supported the reliability of the instrument by demonstrating strong temporal consistency and substantial correspondence between alternate forms. Although the test–retest correlation and ICC indicated good temporal reliability, a small but statistically significant increase in mean scores (1.84 points) was observed over the four-week interval. This systematic increase may reflect a modest practice or familiarity effect resulting from prior exposure to the test rather than true change in nonverbal ability. Therefore, the temporal stability of TONI-3 should be interpreted alongside the possibility of systematic score improvement associated with repeated administration. Criterion-related validity was further supported by strong positive associations with both the NNAT-I and the RSPM, consistent with findings reported in the original test development study (Brown et al., 1997) and previous Turkish validation studies (Bildiren et al., 2024; Korkmaz et al., 2018). Collectively, these findings provide complementary psychometric evidence supporting the reliability and validity of TONI-3 Form A during the middle school period and extend the available Turkish psychometric evidence by integrating both classical and modern psychometric approaches.

The findings of the present study are largely consistent with previous TONI-3 studies conducted with different age groups in Türkiye. In the Turkish validation and standardization study conducted with children aged 6–11 years, TONI-3 demonstrated high internal consistency coefficients and strong correlations with the Raven Standard Progressive Matrices (RSPM) (Korkmaz et al., 2018). Similarly, a reliability and validity study conducted with adolescents aged 14–17 years reported high internal consistency coefficients and strong criterion-related validity with both the RSPM and the Naglieri Nonverbal Ability Test–I (NNAT-I) (Bildiren et al., 2024). These findings are also consistent with other previous Turkish research; for example, the development and revision study of the Nonverbal Ability Test for the Identification Programs of Gifted Students (BNV-II) likewise reported a positive association between BNV-II and TONI-3 (*r* = 0.86), providing additional support for the convergent validity of TONI-3 with other measures of nonverbal reasoning in Turkish samples (Bildiren et al., 2025). Taken together with the findings obtained for the 10 years 0 months to 14 years 0 months age group in the present study, these results provide complementary evidence supporting the reliability and validity of TONI-3 for assessing nonverbal intelligence across a broad developmental period extending from childhood to adolescence. Evidence from international studies is also consistent with the present findings. Research conducted in the United States demonstrated significant associations between TONI-3, the Wechsler Intelligence Scale for Children (WISC), and academic achievement measures, providing evidence of concurrent validity (Banks and Franzen, 2010). Similarly, a Brazilian study involving hearing-impaired students reported age-related improvements in performance together with the expected progression of item difficulty (Barbosa et al., 2013). In addition, Sroythong et al. (2008) found that TONI-3 demonstrated moderate-to-high convergent validity with the Colored Progressive Matrices (CPM) and showed stronger discriminative construct validity than CPM in distinguishing between individuals with different levels of intellectual functioning. Although the strong correlations observed between TONI-3, NNAT-I, and RSPM in the present study provide evidence supporting the criterion-related validity of TONI-3, they should be interpreted with some caution because all three instruments are nonverbal matrix-based measures of reasoning. Consequently, part of the observed associations may reflect shared method variance in addition to the common construct of fluid intelligence. Future studies examining the relationships between TONI-3 and measures of verbal intelligence, general cognitive ability, academic achievement, or other cognitive domains would provide stronger evidence regarding the convergent and discriminant validity of TONI-3. Overall, the available national and international evidence supports the use of TONI-3 as a reliable and valid measure of nonverbal intelligence across different developmental periods and cultural contexts. At the same time, the present findings highlight the importance of extending future validation studies beyond matrix-based nonverbal measures to provide a more comprehensive evaluation of the instrument’s construct validity.

The Rasch analyses also provided evidence regarding the internal structure of TONI-3 Form A. The broad range of item difficulty estimates indicates that the instrument adequately samples different levels of nonverbal ability, while the high Expected A Posteriori (EAP) reliability demonstrates excellent measurement precision. Overall, infit statistics indicated generally acceptable item fit, with the majority of estimable items falling within the commonly recommended range. In contrast, outfit statistics exhibited greater variability, with elevated values observed across both introductory and later items rather than being confined to the easiest basal items. Because outfit statistics are particularly sensitive to unexpected responses on items located far from a respondent’s estimated ability level, they were interpreted cautiously and considered supplementary to the infit statistics, which provided the primary basis for evaluating item fit. Accordingly, the broader distribution of elevated outfit values is more likely to reflect the inherent sensitivity of outfit statistics to such unexpected responses than systematic item misfit.

The CFA findings provided mixed evidence regarding the structural validity of TONI-3 Form A, supporting approximate unidimensionality rather than a strictly uniform one-factor structure. Although the CFI (0.983), TLI (0.982), and RMSEA (0.048) indicated acceptable model fit, the SRMR was elevated (0.141), indicating that the fit of the one-factor model should be interpreted with caution. Accordingly, the CFA findings should be considered alongside the Rasch analyses and local dependence diagnostics, which collectively qualify the interpretation of approximate unidimensionality. A substantial block of the easiest introductory items (Items 2–11) exhibited relatively low standardized factor loadings below 0.40, with several loadings being particularly low (Supplementary Table S1). While very easy basal items are intentionally included in intelligence tests to represent the lower end of the ability continuum and prevent floor effects, these findings indicate that these introductory items contributed limited common-factor information in the present middle-school sample. Therefore, although this pattern is consistent with the intended role of basal items, it should also be considered when interpreting the structural validity evidence for this age group. In contrast to the factor loadings, the estimated threshold values followed the expected ordering across items and were consistent with the hierarchical progression of item difficulty (Supplementary Table S1).

Local dependence diagnostics revealed several item pairs with standardized residual correlations exceeding the reporting criterion of |0.30| (Supplementary Table S2). Several residual correlations approached or exceeded |0.50|, and certain items appeared repeatedly across multiple residual associations. Accordingly, these findings suggest the presence of localized item dependencies. Such residual associations are not uncommon in cognitive ability measures and may reflect similarities in item content or shared solution strategies rather than necessarily indicating additional latent dimensions. Nevertheless, these localized dependencies may have contributed to the elevated SRMR and therefore represent a qualification to the interpretation of the instrument’s approximate unidimensionality. Taken together, the CFA fit indices, Rasch item fit statistics, and local dependence diagnostics provide complementary evidence supporting an interpretation of approximate unidimensionality, although this interpretation is qualified by the residual dependencies, elevated SRMR, and the relatively low factor loadings observed for several introductory items. However, the elevated SRMR, the localized residual dependencies, and the relatively low standardized loadings observed for several introductory items indicate that this interpretation should be made with appropriate caution. Importantly, Item 1 exhibited zero response variability (100% correct response rate) within the present sample, resulting in an extreme Rasch difficulty estimate that was not meaningfully interpretable. This finding likely reflects a ceiling effect for the easiest item rather than a psychometric deficiency of the instrument. Accordingly, Item 1 was excluded from the CFA and DIF analyses, whereas its Rasch difficulty estimate is reported in Supplementary Table S1 for completeness.

To further examine whether the instrument functioned similarly across demographic groups, differential item functioning (DIF) analyses were conducted across gender and age groups (Table 6). Although several items demonstrated nominally significant DIF before adjustment for multiple comparisons, none remained statistically significant after applying the Benjamini–Hochberg false discovery rate correction. Furthermore, the observed item difficulty contrasts for the gender comparison were generally small to modest, providing little evidence of practically meaningful differential item functioning.

Nevertheless, formal measurement invariance analyses were beyond the scope of the present study. The combined findings from the CFA, Rasch, and DIF analyses provide complementary evidence regarding the structural stability of the instrument and the comparability of item functioning across the examined demographic groups. These findings should be interpreted as preliminary evidence and should not be regarded as a substitute for formal measurement invariance testing. Accordingly, formal invariance testing using multi-group CFA or other advanced psychometric approaches is warranted to further evaluate the equivalence of the measurement model across demographic groups. The importance of formal measurement invariance testing has also been demonstrated in recent Turkish research. Bildiren and Akbaş (2025) established scalar measurement invariance for the BNV-I across gender, grade level, age, and ethnic subgroups, indicating that factor loadings and threshold parameters were equivalent across these groups. These findings further underscore the value of formal invariance testing in the validation of nonverbal ability measures and support its inclusion in future psychometric studies of TONI-3.

The findings related to demographic variables showed differences in TONI-3 scores across age and grade groups within the present sample. The significant differences observed across age and grade levels, together with small-to-moderate effect sizes, indicate that older students obtained higher TONI-3 scores than younger students within the present sample. These findings are consistent with previous developmental research reporting positive associations between age and fluid reasoning ability during late childhood and early adolescence (Cattell, 1971; Salthouse et al., 2008). Previous studies have suggested that developmental changes in cognitive processes such as processing speed, working memory, and executive functions may contribute to age-related improvements in nonverbal reasoning (Gray et al., 2017; Cowan et al., 2017). However, because the present study employed a cross-sectional design, these findings should not be interpreted as direct evidence of cognitive maturation or developmental change. Rather, they should be interpreted as descriptive findings indicating that higher TONI-3 scores were observed among older students in the present sample. Furthermore, because formal measurement invariance across demographic groups was not examined, the observed differences should be interpreted with caution, and should not be regarded as definitive evidence of differences in the underlying construct across age or grade groups.

When gender-related findings are considered, although a statistically significant difference favoring female students was observed, the effect size (Cohen’s *d*) was very small. From a practical perspective, this indicates that the magnitude of the observed difference was negligible within the present sample. This finding is broadly consistent with many studies in the literature on nonverbal intelligence. Large-scale studies have frequently reported that measures of nonverbal ability tend to be largely independent of gender. The developers of the test, Brown et al. (1997), did not report significant gender differences during the standardization process. Although the original TONI-3 standardization study did not report significant gender differences, a small but statistically significant difference favoring female students was observed in the present study. This discrepancy may be attributable to differences in sample characteristics, cultural context, or the substantially larger sample size of the present study, which increases statistical power to detect very small effects. Importantly, the observed effect size was negligible (Cohen’s *d* = 0.128), and no meaningful differential item functioning across gender was identified after Benjamini–Hochberg correction. Taken together, these findings suggest that the observed gender difference is unlikely to reflect substantial item-level bias. Nevertheless, because formal measurement invariance across gender was not examined, this finding should be interpreted as descriptive rather than as evidence of true differences in the underlying construct between female and male students. Similarly, studies conducted in Türkiye have shown that TONI-3 scores do not significantly differ by gender in either the 6–11 age group or the 14–17 age group (Korkmaz et al., 2018; Bildiren et al., 2024). This pattern is not limited to TONI-3 but is also observed in other nonverbal assessment instruments (e.g., NNAT-I; CPM) (Bildiren et al., 2017; Kavruk and Bildiren, 2022).

The findings related to SEGE levels indicate that TONI-3 scores were comparable across the district-level socioeconomic development groups represented in the present sample. The absence of statistically significant differences across groups and the negligible effect size are compatible with the intended design of nonverbal ability tests, which seek to reduce the influence of linguistic demands. Accordingly, nonverbal tests based on universal geometric shapes and low-language-load items have been proposed as potentially more equitable assessment tools for individuals from diverse socioeconomic and cultural backgrounds (Bracken and McCallum, 2009; Brown, 2003; Friedlander Moore et al., 2017; McCallum et al., 2001). However, these findings alone do not constitute evidence of cultural or socioeconomic fairness and should be interpreted with caution because SEGE is a district-level indicator rather than an individual-level measure of socioeconomic status. Moreover, the absence of statistically significant differences should not be interpreted as definitive evidence that TONI-3 is independent of socioeconomic influences or that it is inherently culture-fair. Although the present study found no meaningful DIF across age and gender, socioeconomic measurement invariance and DIF across socioeconomic groups were not examined. Therefore, the present findings should be interpreted as preliminary evidence regarding the comparability of TONI-3 performance across the examined groups rather than as definitive evidence of cultural or socioeconomic fairness. Future studies incorporating individual-level socioeconomic indicators together with socioeconomic DIF analyses and formal measurement invariance testing are needed to provide stronger evidence regarding the fairness and cross-group comparability of the instrument.

In summary, the findings provide evidence supporting the use of TONI-3 as a valid nonverbal measure of cognitive ability in middle school students. Furthermore, the absence of meaningful differential item functioning across gender and age groups provides preliminary evidence that item functioning was comparable across the examined demographic groups. However, these findings should not be interpreted as evidence of measurement equivalence in the absence of formal measurement invariance testing. Nevertheless, further research incorporating individual-level socioeconomic indicators together with formal measurement invariance analyses is needed before firm conclusions can be drawn regarding cross-group comparability and the influence of socioeconomic factors on TONI-3 performance.

### 4.1 Limitations

The findings of the present study should be interpreted in light of several limitations. First, the sample consisted solely of middle school students from Aydın province, which may limit the generalizability of the findings to other regions of Türkiye. Second, because the study employed a cross-sectional design, the observed age-related differences cannot be interpreted as evidence of individual developmental change or cognitive maturation. Third, criterion-related validity was examined only through nonverbal matrix-based measures (NNAT-I and RSPM). Accordingly, the observed correlations may partly reflect shared method variance in addition to the common construct of fluid intelligence. The inclusion of measures assessing verbal ability, general cognitive ability, academic achievement, and other cognitive domains would provide a more comprehensive evaluation of the convergent and discriminant validity of TONI-3. Fourth, although the CFA, Rasch, and differential item functioning (DIF) analyses provided complementary evidence regarding the internal structure, measurement precision, and preliminary evidence regarding comparable item functioning of the instrument, formal measurement invariance across demographic groups was beyond the scope of the present study. Accordingly, the observed group differences should be interpreted as descriptive rather than confirmatory until formal measurement invariance has been established. Furthermore, the Rasch analyses were conducted using the one-parameter logistic (Rasch) model, which assumes equal item discrimination across items. Although this assumption is a defining characteristic of the Rasch model, the present study did not formally compare the Rasch model with alternative IRT models that allow item discrimination to vary. Consequently, future studies should examine both formal measurement invariance across demographic groups and the relative fit of alternative IRT models to determine whether the measurement model functions equivalently across populations and whether the assumption of equal item discrimination is appropriate. In addition, socioeconomic status was represented by district-level SEGE classifications rather than individual-level socioeconomic indicators. Therefore, conclusions regarding the socioeconomic fairness of TONI-3 should be interpreted with caution. Future studies should incorporate individual-level socioeconomic variables together with socioeconomic DIF and measurement invariance analyses to provide a more rigorous evaluation of the instrument across socioeconomic groups.

### 4.2 Implications for future research

Future research including participants from different regions of Türkiye would help establish the generalizability of the present findings. Longitudinal studies may provide a clearer understanding of age-related changes in nonverbal intelligence during the middle school years. In addition, formal measurement invariance analyses across age, gender, and socioeconomic groups are needed to determine whether TONI-3 functions equivalently across different subpopulations. Future validation studies would also benefit from incorporating measures of verbal intelligence, general cognitive ability, academic achievement, and behavioral outcomes in order to strengthen the evidence regarding the convergent, discriminant, and criterion-related validity of the instrument.

## 5 Conclusion

The present findings provide evidence supporting the reliability and validity of TONI-3 Form A as a measure of nonverbal intelligence for middle school students ranging in age from 10 years 0 months to 14 years 0 months. By examining this developmental period, the study extends previous Turkish validation research and addresses an important gap in the available psychometric evidence. Findings obtained from classical test theory, together with confirmatory factor analysis (CFA), Rasch model analyses, and differential item functioning (DIF) analyses, provide complementary evidence regarding the measurement precision and item functioning of TONI-3 Form A, whereas the structural analyses yielded mixed evidence supporting approximate unidimensionality rather than a strictly uniform one-factor structure. Accordingly, the combined evidence generally supports the use of TONI-3 Form A as a reliable measure of nonverbal intelligence within the scope of the present study. These findings extend the available psychometric evidence for TONI-3 in Türkiye by integrating classical test theory with contemporary psychometric approaches. Although further research is needed to establish formal measurement invariance across demographic groups, compare alternative IRT models, and evaluate the instrument using a broader range of cognitive measures, the present findings provide preliminary evidence supporting the use of TONI-3 Form A in educational and research settings.

## Data Availability

The datasets generated and/or analyzed during the current study are not publicly available because of institutional and ethical restrictions. Requests for access to the data may be considered by the corresponding author, subject to institutional policies and ethical approval. Requests to access the datasets should be directed to ahmetbildiren@gmail.com.
