# Supplementary material for: Psychometric properties of the test of nonverbal intelligence–third edition (TONI-3) in Turkish middle school students
Source: Front Psychol. 2026 Sep 3;17:1828497. doi: 10.3389/fpsyg.2026.1828497 (PMC13582651; doi:10.3389/fpsyg.2026.1828497)
Supplement: Supplementary file 1 [file Table_1.docx]

**Supplementary Table S1**. Standardized factor loadings, threshold values, and Rasch item statistics for TONI-3 Form A (Items 2–45 for CFA; Items 1–45 for Rasch analysis)

| **Item** | **Standardized factor loading (λ)** | **Threshold (τ)** | **Rasch**  **Difficulty (logits)** | **Proportion Correct** | **Infit MnSq** | **Outfit MnSq** |
| --- | --- | --- | --- | --- | --- | --- |
| Item1 | -- | -- | -40.5 | 1 | -- | --- |
| Item2 | .057 | -2.186 | -5.974 | 0.986 | 1.156 | 38.575 |
| Item3 | .237 | -2.526 | -6.976 | 0.994 | 0.972 | 1.695 |
| Item4 | .157 | -1.805 | -4.898 | 0.964 | 1.349 | 12.629 |
| Item5 | .102 | -2.160 | -5.901 | 0.985 | 1.167 | 19.514 |
| Item6 | .335 | -1.420 | -3.838 | 0.922 | 1.426 | 5.154 |
| Item7 | .191 | -2.472 | -6.811 | 0.993 | 1.035 | 6.22 |
| Item8 | .352 | -2.113 | -5.766 | 0.983 | 1.133 | 2.693 |
| Item9 | .397 | -1.736 | -4.706 | 0.959 | 1.178 | 7.276 |
| Item10 | .345 | -2.213 | -6.052 | 0.987 | 0.981 | 13.497 |
| Item11 | .399 | -1.567 | -4.24 | 0.941 | 1.225 | 4.967 |
| Item12 | .603 | -1.600 | -4.333 | 0.945 | 0.933 | 5.408 |
| Item13 | .444 | -1.627 | -4.406 | 0.948 | 1.163 | 4.456 |
| Item14 | .554 | -1.609 | -4.357 | 0.946 | 0.967 | 7.971 |
| Item15 | .675 | -1.357 | -3.666 | 0.913 | 1.011 | 1.738 |
| Item16 | .649 | -0.812 | -2.188 | 0.792 | 1.133 | 2.111 |
| Item17 | .611 | -0.636 | -1.716 | 0.738 | 1.21 | 1.958 |
| Item18 | .679 | -0.989 | -2.666 | 0.839 | 1.033 | 2.025 |
| Item19 | .619 | -0.769 | -2.073 | 0.779 | 1.147 | 2.782 |
| Item20 | .815 | -1.092 | -2.946 | 0.863 | 0.842 | 4.172 |
| Item21 | .829 | -0.779 | -2.099 | 0.782 | 0.833 | 1.311 |
| Item22 | .564 | 0.016 | 0.023 | 0.494 | 1.366 | 1.685 |
| Item23 | .838 | -0.743 | -2.004 | 0.771 | 0.822 | 1.197 |
| Item24 | .908 | -0.721 | -1.944 | 0.765 | 0.736 | 1.757 |
| Item25 | .797 | -0.335 | -0.911 | 0.631 | 0.916 | 1.503 |
| Item26 | .769 | -0.205 | -0.565 | 0.581 | 0.967 | 1.222 |
| Item27 | .699 | -0.047 | -0.144 | 0.519 | 1.105 | 1.617 |
| Item28 | .701 | 0.200 | 0.517 | 0.421 | 1.137 | 1.307 |
| Item29 | .882 | -0.198 | -0.545 | 0.578 | 0.788 | 0.787 |
| Item30 | .870 | -0.057 | -0.17 | 0.523 | 0.8 | 0.936 |
| Item31 | .730 | 0.420 | 1.114 | 0.337 | 1.123 | 1.129 |
| Item32 | .795 | 0.570 | 1.524 | 0.284 | 0.954 | 0.742 |
| Item33 | .864 | 0.335 | 0.882 | 0.369 | 0.803 | 0.659 |
| Item34 | .802 | 0.731 | 1.967 | 0.232 | 0.935 | 0.651 |
| Item35 | .850 | 0.753 | 2.029 | 0.226 | 0.841 | 0.544 |
| Item36 | .937 | 0.517 | 1.378 | 0.303 | 0.695 | 0.472 |
| Item37 | .896 | 0.877 | 2.373 | 0.19 | 0.75 | 0.42 |
| Item38 | .927 | 0.909 | 2.463 | 0.182 | 0.678 | 0.344 |
| Item39 | .962 | 0.888 | 2.403 | 0.187 | 0.623 | 0.321 |
| Item40 | .863 | 1.170 | 3.186 | 0.121 | 0.837 | 0.362 |
| Item41 | .898 | 1.114 | 3.032 | 0.133 | 0.759 | 0.33 |
| Item42 | .781 | 1.542 | 4.217 | 0.061 | 0.908 | 0.321 |
| Item43 | .848 | 1.504 | 4.111 | 0.066 | 0.815 | 0.247 |
| Item44 | .708 | 1.977 | 5.424 | 0.024 | 0.944 | 0.25 |
| Item45 | .853 | 1.609 | 4.401 | 0.054 | 0.846 | 0.233 |

**Note.** Standardized factor loadings are reported as completely standardized estimates (Std.all) from the one-factor CFA model estimated using the DWLS estimator. Threshold values represent the estimated threshold for each dichotomously scored item. Item 1 was answered correctly by all participants (100% correct response rate), resulting in zero variance. Consequently, it was excluded from the CFA and DIF analyses. In the Rasch analysis, Item 1 was retained in the calibration and in the estimation of EAP person reliability. It yielded an extreme difficulty estimate (−40.50 logits), which is reported for completeness but should not be interpreted as a meaningful item difficulty estimate. Therefore, the reported interpretable item difficulty range refers to Items 2–45.

**Supplementary Table S2.** Standardized residual correlations (absolute values ≥ .30) from the local dependence diagnostics of the one-factor CFA model.

| **Item 1** | **Item 2** | **Standardized residual correlation** |
| --- | --- | --- |
| Item 14 | Item 3 | 0.550 |
| Item 44 | Item 20 | −0.542 |
| Item 44 | Item 14 | −0.481 |
| Item 13 | Item 3 | 0.467 |
| Item 10 | Item 2 | 0.463 |
| Item 18 | Item 3 | 0.460 |
| Item 20 | Item 3 | 0.457 |
| Item 3 | Item 2 | 0.450 |
| Item 10 | Item 8 | 0.442 |
| Item 14 | Item 10 | 0.438 |
| Item 42 | Item 12 | −0.433 |
| Item 5 | Item 3 | 0.428 |
| Item 11 | Item 7 | 0.428 |
| Item 13 | Item 10 | 0.424 |
| Item 12 | Item 3 | 0.420 |
| Item 43 | Item 20 | −0.403 |
| Item 42 | Item 14 | −0.399 |
| Item 10 | Item 3 | 0.393 |
| Item 44 | Item 3 | −0.387 |
| Item 43 | Item 24 | −0.385 |
| Item 45 | Item 20 | −0.383 |
| Item 45 | Item 14 | −0.381 |
| Item 7 | Item 3 | 0.378 |
| Item 14 | Item 2 | 0.376 |
| Item 17 | Item 3 | 0.374 |
| Item 43 | Item 12 | −0.367 |
| Item 42 | Item 20 | −0.367 |
| Item 39 | Item 12 | −0.362 |
| Item 9 | Item 7 | 0.361 |
| Item 14 | Item 12 | 0.354 |
| Item 23 | Item 3 | 0.352 |
| Item 45 | Item 10 | −0.351 |
| Item 8 | Item 3 | 0.350 |
| Item 39 | Item 24 | −0.345 |
| Item 10 | Item 9 | 0.344 |
| Item 42 | Item 24 | −0.343 |
| Item 40 | Item 14 | −0.339 |
| Item 36 | Item 12 | −0.336 |
| Item 12 | Item 11 | 0.334 |
| Item 41 | Item 14 | −0.334 |
| Item 43 | Item 14 | −0.330 |
| Item 44 | Item 7 | −0.327 |
| Item 39 | Item 14 | −0.321 |
| Item 15 | Item 3 | 0.317 |
| Item 39 | Item 20 | −0.314 |
| Item 44 | Item 10 | −0.314 |
| Item 38 | Item 14 | −0.311 |
| Item 41 | Item 12 | −0.310 |
| Item 43 | Item 10 | −0.308 |
| Item 38 | Item 12 | −0.300 |
| Item 13 | Item 2 | 0.300 |

**Note.** Standardized residual correlations were obtained from the ordinal one-factor CFA model estimated using the diagonally weighted least squares (DWLS) estimator. Only item pairs with absolute residual correlations ≥ |0.30| are reported. Positive values indicate greater-than-expected residual associations, whereas negative values indicate lower-than-expected residual associations after accounting for the common factor.
